# Factors Associated with Treatment Outcome of Preterm Babies at Discharge from the Neonatal Intensive Care Unit (NICU) of the Tamale Teaching Hospital, Ghana

**DOI:** 10.1155/2020/5696427

**Published:** 2020-08-14

**Authors:** Alhassan Abdul-Mumin, Sheila Agyeiwaa Owusu, Abdulai Abubakari

**Affiliations:** ^1^Department of Paediatrics and Child Health, School of Medicine and Health Sciences, University for Development Studies, Tamale, Ghana; ^2^Department of Paediatrics and Child Health, Tamale Teaching Hospital, Tamale, Ghana; ^3^Department of Public Health, School of Allied Health Sciences, University for Development Studies, Tamale, Ghana

## Abstract

**Background:**

Preterm birth and complications are now the leading cause of death in children under 5 years globally. In Ghana, studies assessing the survival rate of preterm babies and associated factors in Neonatal Intensive Care Units (NICU) are limited. Therefore, this study was designed to assess the survival rate and associated factors in this group of babies in a teaching hospital in the Northern Region of Ghana.

**Methods:**

This was a 7-month retrospective descriptive study conducted in the NICU of the Tamale Teaching Hospital, Ghana. It involved review of charts of all preterm babies admitted between 1 March 2017 and 30 September 2017. Data retrieved from all eligible patients was analyzed using Stata version 12.1 software to generate descriptive statistics. Relationship between dependent and independent variables was tested using Pearson chi square. A logistic regression model was estimated to assess determinants of the treatment outcome.

**Results:**

The overall survival rate at discharge in this cohort was 60.73%. The survival rate was lowest in the extremely low birth weight group (3/21; 14.3%) and extremely preterm babies (4/20; 20%). Significant association was observed between birth weight (*P* = 0.0001), gestational age (*P* = 0.0001), and survival. Preterm babies who were hypothermic at presentation, had respiratory distress syndrome, and had jaundice were 7.2 times (AOR = 7.2; 95%CI = 1.9‐28.1; *P* = 0.004), 10.2 times (AOR = 10.2; 95%CI = 3.7‐27.9; *P* ≤ 0.0001), and 2.9 times (AOR = 2.9; 95%CI = 1.0‐8.5; *P* = 0.045), respectively, more likely to die on admission compared to neonates who did not have these comorbidities.

**Conclusion:**

We found a high mortality rate in the preterm babies admitted to our unit, and that mortality rate decreased with increasing gestational age and birth weight. A number of neonatal factors, either in isolation or in combination, were significantly associated with in-hospital mortality.

## 1. Introduction

Newborn babies in need of critical medical attention are normally admitted to the Neonatal Intensive Care Unit (NICU) [1]. These infants may be preterm (i.e., born before 37 completed weeks of pregnancy) and have a low birth weight and/or serious medical condition [1].

Preterm birth is defined by the World Health Organization (WHO) as all births before 37 completed weeks of gestation or fewer than 259 days since the first day of a woman's last menstrual period [2].

Being born preterm also increases a baby's risk of dying due to other causes, especially from neonatal infections with preterm birth estimated to be a risk factor in at least 50% of all neonatal deaths [3].

More than 1 in 10 of the world's babies born in 2010 were born prematurely [4], and more than 1 million of the estimated 15 million preterm births died as a result of their prematurity [5]. Preterm birth complications are now the leading cause of death in children under 5 years and the single most important cause of death in the critical first month of life [6].

The WHO classification categorizes preterm births into extreme preterm (<28 completed weeks), very preterm (28-<32 completed weeks), and moderate to late preterm (32-<37 completed weeks) [7]. According to WHO, over 90% of the extremely preterm babies (<28 weeks) born in low-income countries die within the first few days of life; yet, less than 10% of babies of this gestation die in high-income settings, a 10 : 90 survival gap [7].

The outcome of preterm babies is assessed as survival of the preterm baby and short- and long-term morbidity associated with preterm delivery. This is usually dependent on a number of factors such as biologic maturity and technological factors [6]. The survival of these babies has continued to improve in most developed countries, with continuing progress in neonatal intensive care, shifting the limit of viability towards younger gestational ages, with greater than 80% survival at 28 weeks' gestation [4, 6]. The same cannot be said for most African countries with poor health care infrastructure, heavy debt burden, conflicts, and endemic poverty. In Nigeria, survival below 28 weeks of gestation is less than 20% [8].

Neonatal survival varies with the quality of medical care [9]. In developed countries, newborns typically die from unpreventable causes, such as congenital malformations, whereas the majority of infants in developing countries die from preventable conditions, including infections, birth asphyxia, and prematurity [10].

Studies have found that primary causes of death for 78% of all non-malformation-related deaths in preterm babies were severe perinatal asphyxia, respiratory distress syndrome (RDS), and infection. Other causes of death were bronchopulmonary dysplasia (4%), necrotizing enterocolitis (2%), intraventricular hemorrhage (3%), and pneumothorax (2%) [11, 12].

In Ghana, studies assessing the survival rate of premature babies and associated factors in the NICU are limited. Therefore, this study was designed to assess the survival rate of this group of neonates and associated factors in the Tamale Teaching Hospital (TTH) in the Northern Region of Ghana.

## 2. Materials and Methods

### 2.1. Study Site

The TTH is a 450-bed capacity hospital which serves as the only teaching hospital in the northern part of Ghana. It is the main center for clinical training of medical students from the University for Development Studies (UDS), Tamale, Ghana. The hospital has a 40-incubator/crib capacity NICU with a 5-bed Kangaroo Mother Care (KMC) unit attached to it. The unit is able to provide respiratory support through a bubble Continuous Positive Airway Pressure (CPAP) setup. It is unable to provide invasive ventilation. With majority of its patients coming from the Tamale Metropolis, the approximate population of the catchment area is about 4 million.

### 2.2. Study Design

This was a 7-month retrospective descriptive study conducted in the NICU of the TTH. It involved review of charts of all preterm babies admitted between 1 March 2017 and 30 September 2017.

### 2.3. Patients and Methods

A hospital record officer was assigned to retrieve case files of all preterm babies admitted to the NICU during the study period. The records retrieved were retrospectively reviewed for birth weight, gestational age, sex, mode of delivery, place of delivery, length of stay, and outcome of admission. Maternal information including age, parity (number of pregnancies carried to at least 24 weeks of gestational age), and illness in pregnancy was also retrieved.

### 2.4. Inclusion Criteria

All preterm babies admitted at the TTH NICU during the study period (1 March 2017 to 30 September 2017) were eligible for inclusion in the study. The participants included both inborn and outborn babies.

### 2.5. Exclusion Criteria

We excluded babies whose folders could not be retrieved from the records and low birth weight babies who were born at term.

### 2.6. Data Analysis

The data were entered into Excel and exported into Stata (V12.1). The treatment outcome was categorized into discharged and died on admission while birth weight and gestational age at birth were categorized using WHO standards. The explanatory variables included gestational age at birth, comorbidities at presentation (RDS, hypothermia, hypoglycemia, neonatal jaundice, and sepsis), sex, mode of delivery, and birth weight. Gestational age was available for 187 participants while 161 participants had their birth weight data recorded. Therefore, analysis with respect to gestational age and birth weight was conducted on only 187 and 161 participants, respectively. The first part of the analysis dealt with descriptive statistics to ascertain the frequency of basic infant, maternal, and obstetric characteristics and to screen all the potential independent variables individually for their relationship with the dependent variable using a chi-squared test. In other words, the chi-squared test was used to establish if there was any association between basic infant, maternal, and obstetric characteristics and treatment outcome. Bonferroni's correction was applied to correct for multiple testing. The chi-squared test is a nonparametric test that is appropriate for examining whether there is a significant relationship between nominal categorical variables. The test is based on the crosstabulation of the relevant variables and compares the observed frequencies in each cell of the crosstabulation to the frequencies expected if there were no relationship between the variables [13]. A chi-squared test was used in this analysis because each observation was independent of all others and no more than 20% of the expected counts were less than five [14]. The influence of the independent variables on the dependent variable (treatment outcome) was determined using multiple logistic regression, producing adjusted odds ratios (AOR) that control for other predictor variables in the multiple regression model, and 95% confident intervals (CI). Multicollinearity and independence of observations which are necessary conditions for multiple logistic regression [15] were checked. The treatment outcome was analyzed as a binary variable.

All independent variables with *P* values less than 0.1 in the chi-squared analysis were included in the final logistic regression model. *P* value < 0.05 at 95% CI was considered statistically significant.

### 2.7. Ethical Clearance

Ethical clearance for this study was obtained from the TTH Ethical Review Committee with clearance no.: TTHERC/20/04/18/02.

## 3. Results

A total of 192 preterm babies were included in the study. This translated to 19.2% (192/1000) of the total admissions to the unit during the study period.

Of the babies with a birth weight documented at admission, 13.04% have extremely low birth weight and 40.99% have very low birth weight while 45.34% have moderately low birth weight. About 10.7% of the babies were extreme preterm and 31.02% were very preterm while 58.29% were moderate to late preterm according to the WHO classifications. Out of the total records, 52.13% were female while 47.87% were males. Over 55% were born through spontaneous vaginal delivery (SVD) compared to about 44.8% who were born via cesarean section (C/S).  Table 1 shows details of the baseline characteristics of the babies included in the study.

Table 2 shows the comorbidities documented in our patients at presentation. In the majority, 67.7% were hypothermic, about 13% were hypoglycemic, 11.5% had sepsis, and 43.23% had RDS (Table 2).

### 3.1. Factors Associated with Treatment Outcome of Preterm Babies

The overall survival rate at discharge in this cohort was 60.73%. The survival rate was lowest in the extremely low birth weight group (14.3%) and highest in the moderately low birth weight group (82.2%). The survival rate was also the lowest among the extremely preterm babies (gestational age < 28 weeks) (20%) compared to the moderate to late preterm babies (75.0%). Survival was lower among preterm babies admitted in the first 3 days of life (58.04%) compared to older babies (80.0%). In the bivariate analysis, neonates with birth weight < 1000 g (85.7%) and those with birth weight between 1000 g and 1499 g (5%) had significantly higher mortalities (*P* ≤ 0.0001). Similarly, those with gestational age of <28 weeks (80.0%) and 28-<32 weeks also had significantly higher mortalities (*P* ≤ 0.0001). This implies that gestational age and birth weight at birth were statistically significantly associated with the treatment outcome.  Table 3 shows the details of the baseline characteristics and their association with the treatment outcome of preterm babies.

Among the comorbidities identified at presentation, those presenting with hypothermia were more likely to die (50.0%) compared to those who were not (16.4%). Moreover, those presenting with RDS were also more likely to die (72.3%) compared to those without this diagnosis (13.9%). Further analysis of the data revealed that neonates who were hypothermic and at the same time hypoglycemic at the time of admission were also more likely to die (68.42%) compared to neonates who were either hypothermic or hypoglycemic (36.05%). Further, neonates who had both RDS and hypothermia were also more likely to die (73.08%) than those who had either RDS or hypothermia (33.94%).  Table 4 shows the details of the association between the various comorbidities and treatment outcomes.

In the bivariate analysis, patients with hypothermia (*P* = 0.0001), RDS (*P* = 0.0001), hypothermia and hypoglycemia combined (*P* = 0.006), and RDS together with hypothermia (*P* = 0.0001) had a statistically significant less favorable outcome (Table 4) compared to babies who did not have these comorbidities.

In the multiple logistic regression analysis (Table 5), neonates who were hypothermic at presentation were about 7.2 times more likely to die on admission compared to those who were not hypothermic at presentation (AOR = 7.2; 95%CI = 1.9‐28.1; *P* = 0.004). Moreover, neonates who had RDS at presentation were about 10.2 times more likely to die on admission compared to those who did not have RDS at presentation (AOR = 10.2; 95%CI = 3.7‐27.9; *P* ≤ 0.0001). Finally, preterm babies with jaundice diagnosis were also 2.9 times more likely to die on admission compared to those who had no jaundice at presentation (AOR = 2.9; 95%CI = 1.0‐8.5; *P* = 0.045) (Table 5).

## 4. Discussion

Neonatal mortality attributable to preterm birth and complications remains a huge challenge globally and in low- and middle-income countries like Ghana. A few studies in Ghana have looked at predischarge mortality in this group of neonates [16]. This retrospective hospital-based study was therefore designed to assess the predischarge survival and factors associated with it in preterm babies admitted to the NICU of a teaching hospital in the Northern Region of Ghana.

Many of the patients in our cohort were born with extremely or very low birth weight and were in the gestational age ranges categorized by WHO as extremely or very preterm [7]. These two factors have been well studied in previous studies to affect outcomes of preterm babies in different countries [8, 16]. Many of the complications of preterm birth that lead to adverse in-hospital outcomes depend on the gestational age, with more premature infants being prone to more complications.

We documented an overall mortality rate of about 39% in this cohort prior to discharge from the NICU during the study period. Our mortality was comparable to the preterm-specific mortality rate reported in a study conducted at the NICU of the Korle BU Teaching Hospital in Accra, Ghana [16].

In another study conducted in the Mother-Baby Unit of the Komfo Anokye Teaching Hospital, Kumasi, Ghana [17], the survival rates in the preterm baby group were about 68%.

Similarly, high preterm-specific mortality has been documented in countries across sub-Saharan Africa [10] and some other low- and middle-income countries outside Africa. The mortality rate found in the present study was much higher than that in developed countries such as Israel (0.2%) between 2000 and 2009 [18], Canada (7.6%) between 1988 and 2007 [19], and South Africa (3.1-3.4%) from 2007 to 2008 [20]. The similarities found in the survival rates in our study and previous studies from low- and middle-income countries and the difference between it and other studies from more developed settings could be partly explained by the weaker health care systems in our setting and its impact on the quality of care provided during the perinatal period.

Among the neonatal factors we assessed, lower gestational age, lower birth weight, RDS, presence of jaundice, and hypothermia at presentation to the NICU were significantly associated with death in the bivariate analysis. Neonates who had more than one of these risk factors were more likely to die than those who have none or just one risk factor (Table 4). Some of these findings have been documented in previous studies [10, 17].

The association between lower gestational age and mortality was significant in bivariate analysis (*P* = 0.0001). In the present, the study survival rate below 28 weeks of gestation was 20% which is close to the less than 20% found in Nigeria for 27-28 weeks of gestation in 2010 [9]. Gestational age is a key factor in the survival of preterm babies worldwide. This is likely due to the maturity of the lungs as the pregnancy progresses with births at lower gestational age more likely to require surfactant treatment and respiratory support, not readily available in resource-limited settings like ours. It is well known that survival of preterm neonates increases with each additional gestational week they spend in utero [21, 22]. It may therefore be in line with this that we found in our study a significant relationship between mortality and RDS in both the bivariate (*P* = 0.001) and multiple logistic regression (*P* = 0.0001) analyses. Our center is unable to deliver surfactant therapy and only provide bubble CPAP for babies requiring respiratory support [23].

Low birth weight generally accompanies preterm births. In our cohort, >99% (Table 2) of the patients weighed <2500 g at birth. The survival rates of the extremely low birth weight (≤1000 g, <15%) and very low birth weight neonates (1000-1500 g, 50%) were especially low.

Compared to a previous study in Accra, Ghana, our weight-specific survival rate was similar for the extremely low birth weight group, but lower for the very low birth weight group [16].

Other studies in the African region have also found low survival rates for this group of preterm babies [24]. Studies in the developed world have shown much higher survival rates for extremely and very low birth weight neonates. In the United States, one study in the extremely low birth weight babies admitted found a mortality of 34% between 2006 and 2009 [25]; 45.9% was found in India [26] and 46% in Australia [27], which are lower than the mortality (85.7%) found in the present study. Besides this, studies on mortality among neonates with very low birth weight also found an in-hospital mortality rate of 5% in New Zealand in 2009 [28], 6.5% in Korea in 2009 [29], and 12.9% in the United States of America between 2007 and 2008 [30], which are all much lower than the mortality rate found in our study. Previous studies have attributed the chances of survival of preterm babies to a function of biological maturity and technological advancement [31, 32]. We also think especially that the technological advancement explains why viability and survival at lower gestational age and birth weights have improved so much in the developed world compared to our setting.

Hypothermia was common at presentation to our NICU (Table 2) during the study period, and we showed in both the bivariate (*P* = 0.001) and multiple logistic regression (*P* = 0.008) analyses that babies with hypothermia at the time of admission were significantly likely to die compared to those who had normal body temperature (Tables 4 and 5). In fact, in the multiple logistic regression analysis, the babies with hypothermia at presentation were about 6 times more likely to die on admission. Previous studies have found a correlation between lower admission temperature and mortality in preterm babies [33, 34]. One study also assessed and documented a relationship between lower temperature at admission and serious comorbidities like retinopathy, necrotizing enterocolitis, and bronchopulmonary dysplasia [33]. Achieving optimal thermal control is a big challenge for preterm neonates, and hypothermia complicates many of the common comorbidities (hypoglycemia, sepsis, and RDS) observed in preterm babies resulting in adverse outcomes if not managed well. We found in this study that the patients with both hypothermia and hypoglycemia (*P* = 0.006) and those with both hypothermia and RDS (*P* = 0.001) were significantly more likely to die on admission. Hence, it is important to observe and adhere strictly to the warm chain protocol across the continuum of care in order to reduce the risk of babies developing these conditions associated with hypothermia.

Our study could not find a significant relationship between sepsis, mode of delivery, age at presentation, maternal age, and risk of death.

We did not do routine blood cultures for all babies admitted with prematurity. This could limit our ability to identify many of the babies with accompanying sepsis as signs and symptoms of sepsis may mimic many of the features of prematurity. We acknowledge this as one of the limitations of the study.

As a retrospective hospital-based study, our study had a number of limitations. One of them was the fact that our analysis was limited to the information found in the patient records we were able to retrieve. This could have introduced bias in the sampling. Our findings may also represent the profile of patients we admit and may not be generalizable to other units. We also mainly assessed neonatal risk factors, except the maternal age. The fact that our unit is unable to do routine blood cultures means our diagnosis of sepsis may be an underestimation of the problem. Despite this, our findings were similar to those from other studies on the same topic in similar settings, and we think it serves as a baseline for more comprehensive studies to be conducted in the future.

In conclusion, we found a high mortality rate in the preterm babies admitted to our unit and that mortality decreased with increasing gestational age and birth weight. We also found that a number of neonatal factors, either in isolation or in combination, were significantly associated with in-hospital mortality. The fact that there are much improved outcomes in more developed countries means that there is an opportunity to change the narrative for this vulnerable group by improving the quality of care provided during the antenatal and perinatal periods. In line with this high burden of hypothermia, we recommend routine feedback to all referring facilities and units, especially regarding maintenance of the warm chain during transport. Further studies are required to document the maternal risk factors associated with preterm birth and mortality in our hospital.

## Figures and Tables

**Table 1:** Baseline characteristics of preterm babies at presentation to the NICU.

Variable	Categories	*n*/*N*^∗^ (%)
Birth weight (g)	<1000	21/161 (13.04)
	1000-1499	66/161 (40.99)
	1500-2499	73/161 (45.34)
	≥2500	1/161 (0.62)
Gestational age (weeks)	<28	20/187 (10.70)
	28-<32	58/187 (31.02)
	32-37	109/187 (58.29)
Sex	Female	98/188 (52.13)
	Male	90/188 (47.87)
Mode of delivery	C/S	82/189 (44.8)
	SVD	105/189 (55.2)
Age on admission (days)	1-3	144/181 (74.56)
	4-7	12/181 (6.63)
	>7	25/181 (13.81)

^∗^N = the total sample; *n* = the subsample.

**Table 2:** Comorbidities of preterm babies at presentation to the NICU.

Variable	Categories	*n*/*N*^∗^ (%)
Sepsis	Yes	22/192 (11.46)
	No	170/192 (88.54)
Hypothermia	Yes	130/192 (67.71)
	No	62/192 (32.29)
Hypoglycemia	Yes	25/192 (13.02)
	No	167/192 (86.98)
RDS	Yes	83/192 (43.23)
	No	109/192 (56.77)
Neonatal jaundice	Yes	63/192 (32.81)
	No	129/192 (67.19)

^∗^N = the total sample; *n* = the subsample.

**Table 3:** Bivariate analysis of baseline maternal and neonatal characteristics at presentation.

Variable	Categories	Discharged	Died	*P* value
		*n*/*N*^∗^ (%)	*n*/*N* (%)	
Birth weight (g)	<1000	3/21 (14.3)	18/21 (85.7)	<0.0001
	1000-1499	33/66 (50.0)	33/66 (50.0)	
	1500-2499	60/73 (82.2)	13/73 (17.8)	
Gestational age (weeks)	<28	4/20 (20.0)	16/20 (80.0)	<0.0001
	28-<32	27/58 (46.55)	31/58 (53.45)	
	32-37	81/108 (75.0)	27/108 (25.0)	
Sex	Male	57/89 (64.0)	32/89 (36.0)	0.410
	Female	57/98 (58.2)	41/98 (41.8)	
Age on admission (days)	1-3	83/143 (58.04)	60/143 (41.96)	0.072
	4-7	9/12 (75)	3/12 (25)	
	>7	20/25 (80)	5/25 (20)	
Mode of delivery	C/S	58/82 (70.7)	24/82 (29.3)	0.078
	SVD	57/101 (56.4)	44/101 (43.6)	
Maternal age (years)	≤20	14/17 (82.3)	3/17 (17.7)	0.168
	21-30	59/94 (62.8)	35/94 (37.2)	
	≥31	26/46 (56.5)	20/46 (43.48)	

^∗^N = number of patients in a category; *n* = number in a category who died or were discharged.

**Table 4:** Bivariate analysis of association between treatment outcome and comorbidities at presentation.

Variable	Categories	Discharged	Died	*P* value
		*n*/*N*^∗^ (%)	*n*/*N* (%)	
Sepsis	Yes	13/22 (59.1)	9/22 (40.9)	0.867
	No	103/169 (60.9)	66/169 (39.1)	
Hypothermia	Yes	65/130 (50.0)	65/130 (50.0)	0.001
	No	51/61 (83.6)	10/61 (16.4)	
Hypoglycemia	Yes	13/25 (52.0)	12/25 (48.0)	0.338
	No	103/166 (62.1)	63/166 (37.9)	
RDS	Yes	23/83 (27.7)	60/83 (72.3)	0.001
	No	93/108 (86.1)	15/108 (13.9)	
Neonatal jaundice	Yes	49/63 (77.8)	14/63 (22.2)	0.001
	No	67/128 (52.3)	61/128 (47.7)	
Hypothermia & hypoglycemia	Yes	6/19 (31.58)	13/19 (68.42)	0.006
	No	119/172 (63.95)	62/172 (36.05)	
RDS & hypothermia	Yes	7/26 (26.92)	19/26 (73.08)	0.001
	No	109/165 (66.06)	56/165 (33.94)	

^∗^N = number of patients in a category; *n* = number in a category who died or were discharged.

**Table 5:** Multiple logistic regression analysis of determinants of treatment success of preterm babies.

Variables	Variable categories	AOR (95% CI)	*P* value
Hypothermia	Yes	7.2 (1.9-28.1)	0.004
	No	Reference	
RDS	Yes	10.2 (3.7-27.9)	<0.0001
	No	Reference	
Birth weight (g)	<1000	0.6 (0.04-8.1)	0.668
	1000-1499	0.4 (0.03-3.2)	0.371
	1500-2499	Reference	
Gestational age (weeks)	<28	1.5 (0.2-10.3)	0.668
	28-<32	0.8 (0.2-4.3)	0.803
	32-37	Reference	
Neonatal jaundice	Yes	2.9 (1.0-8.5)	0.045
	No	Reference	
Mode of delivery	C/S	1.6 (0.6-4.6)	0.384
	SVD	Reference	
Age on admission (days)	1-3	6.0 (0.6-63.2)	0.134
	4-7	1.6 (0.1-33.4)	0.763
	>7	Reference	

## Data Availability

The data supporting the conclusion of this paper are included within the manuscript. Upon reasonable request, the dataset could be obtained from the corresponding author.

## Abbreviations

AOR: Adjusted odds ratio
CPAP: Continuous Positive Airway Pressure
C/S: Cesarean section
NICU: Neonatal Intensive Care Unit
RDS: Respiratory distress syndrome
SVD: Spontaneous vaginal delivery
TTH: Tamale Teaching Hospital
WHO: World Health Organization.
